# Clinical Characteristics of *POC1B*-Associated Retinopathy and Assignment of Pathogenicity to Novel Deep Intronic and Non-Canonical Splice Site Variants

**DOI:** 10.3390/ijms22105396

**Published:** 2021-05-20

**Authors:** Nicole Weisschuh, Pascale Mazzola, Miriam Bertrand, Tobias B. Haack, Bernd Wissinger, Susanne Kohl, Katarina Stingl

**Affiliations:** 1 Centre for Ophthalmology, Institute for Ophthalmic Research, University of Tübingen, 72070 Tübingen, Germany; nicole.weisschuh@uni-tuebingen.de (N.W.); wissinger@uni-tuebingen.de (B.W.); susanne.kohl@uni-tuebingen.de (S.K.); 2 Institute of Medical Genetics and Applied Genomics, University of Tübingen, 72070 Tübingen, Germany; pascale.mazzola@med.uni-tuebingen.de (P.M.); miriam.bertrand@med.uni-tuebingen.de (M.B.); tobias.haack@med.uni-tuebingen.de (T.B.H.); 3 Centre for Rare Diseases, University of Tübingen, 72070 Tübingen, Germany; 4 Centre for Ophthalmology, University Eye Hospital, University of Tübingen, 72070 Tübingen, Germany

**Keywords:** cone dystrophy, cone-rod dystrophy, *POC1B*, non-canonical splice site variant, deep intronic splice variant, in vitro splice assay, multimodal phenotyping

## Abstract

Mutations in *POC1B* are a rare cause of inherited retinal degeneration. In this study, we present a thorough phenotypic and genotypic characterization of three individuals harboring putatively pathogenic variants in the *POC1B* gene. All patients displayed a similar, slowly progressive retinopathy (cone dystrophy or cone-rod dystrophy) with normal funduscopy but disrupted outer retinal layers on optical coherence tomography and variable age of onset. Other symptoms were decreased visual acuity and photophobia. Whole genome sequencing revealed a novel homozygous frameshift variant in one patient. Another patient was shown to harbor a novel deep intronic variant in compound heterozygous state with a previously reported canonical splice site variant. The third patient showed a novel nonsense variant and a novel non-canonical splice site variant. We aimed to validate the effect of the deep intronic variant and the non-canonical splice site variant by means of in vitro splice assays. In addition, direct RNA analysis was performed in one patient. Splicing analysis revealed that the non-canonical splice site variant c.561-3T>C leads to exon skipping while the novel deep intronic variant c.1033-327T>A causes pseudoexon activation. Our data expand the genetic landscape of *POC1B* mutations and confirm the benefit of genome sequencing in combination with downstream functional validation using minigene assays for the analysis of putative splice variants. In addition, we provide clinical multimodal phenotyping of the affected individuals.

## 1. Introduction

The 12 exons of the *POC1B* transcription unit (GenBank NM_172240.3, OMIM * 614784) span 106.3 kb on chromosome 12q21.33 and code for one of the two POC1 proteins found in humans. POC1B acts together with POC1A to maintain centriole integrity and proper mitotic spindle formation [1,2]. In the human retina, *POC1B* is predominantly expressed in the ciliary region of photoreceptors and at the synapses of the outer plexiform layer [3]. Mutations in *POC1B* were first discovered to cause autosomal-recessive cone-rod dystrophy (CORD) [3,4]. Subsequently, mutations in *POC1B* have been found in patients with Leber congenital amaurosis with features of a syndromic ciliopathy [5], and in patients with cone dystrophy (CD) [6,7]. The retinopathy caused by recessive biallelic mutations in *POC1B* shows several phenotypic characteristics. Most patients harboring pathogenic variants in *POC1B* have normal funduscopic appearance and normal rod function [4,6,7]. However, CORD and funduscopic abnormalities have also been described [3], as has progression from CD to CORD [8]. Of note, though funduscopic appearance appears normal, changes of the outer retina are visible in optical coherence tomography (OCT), displaying a disruption of ellipsoid zone (EZ) and outer/inner segment lines [4,7,9,10], leading to impaired visual acuity and photophobia.

To date, the Human Gene Mutation Database (HGMD) [11] lists only nine *POC1B* mutations, comprising five missense, two nonsense, one in frame deletion, and one canonical splice site variant. In addition, two non-canonical splice site (NCSS) variants were found in compound heterozygous state in a patient diagnosed with CORD [12].

As mutations in *POC1B* are a rare cause of inherited retinal degeneration, we aimed to characterize three patients with biallelic *POC1B* variants at the clinical and molecular level.

## 2. Results

### 2.1. Clinical Phenotype

**Figure 1:**
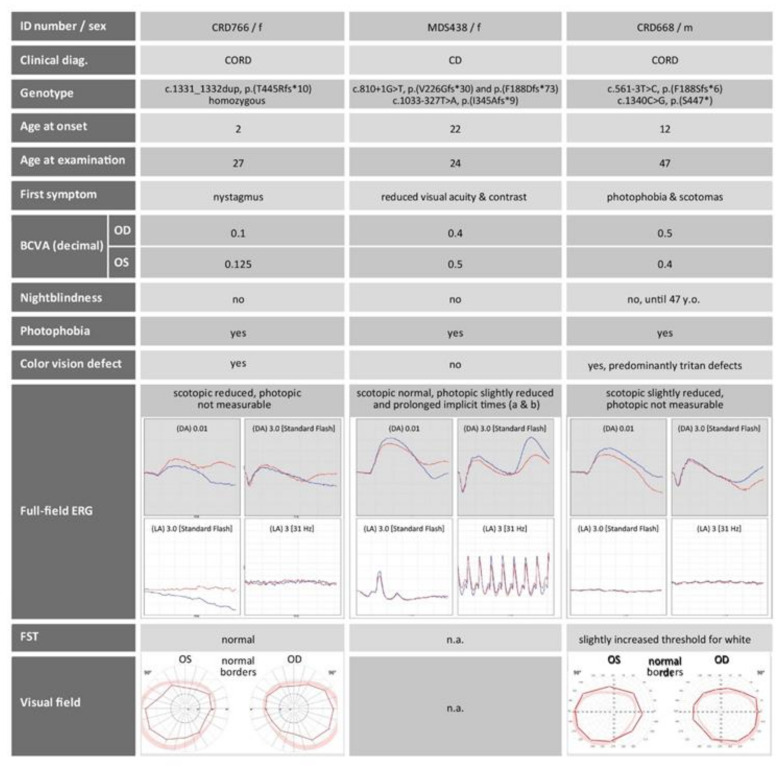
Medical history and retinal functional diagnostics of the three patients described in this study. F, female; m, male; CORD, cone-rod dystrophy; CD, cone dystrophy; OD, right eye; OS, left eye; n.a., not available; BCVA, best corrected visual acuity; DA, dark adapted; LA, light adapted; FST, full-field stimulus threshold. Age is given in years. Mutation nomenclature refers to *POC1B* with GenBank accession number NM_172240.3.

**Figure 2:**
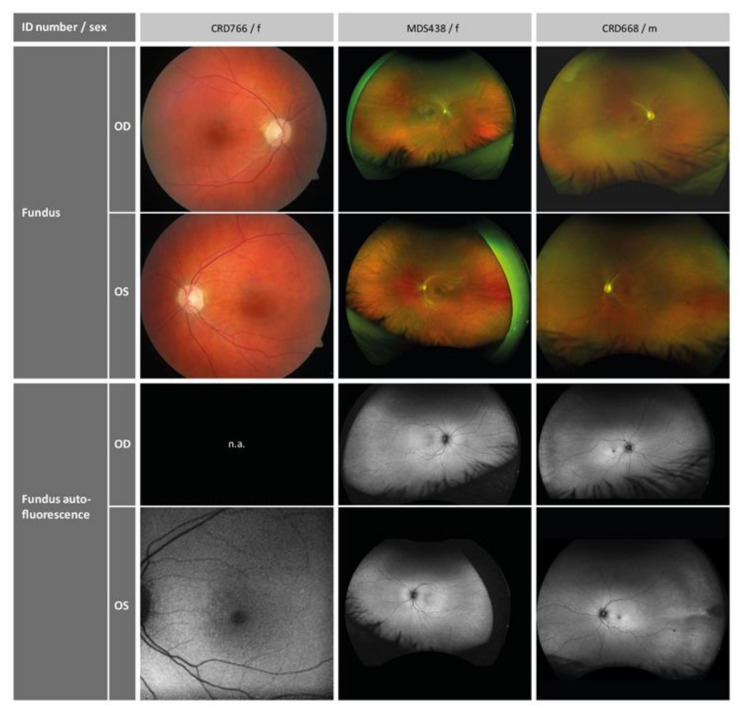
Retinal imaging of the three patients. OD, right eye; OS, left eye; f, female; m, male; n.a., not available.

**Figure 3:**
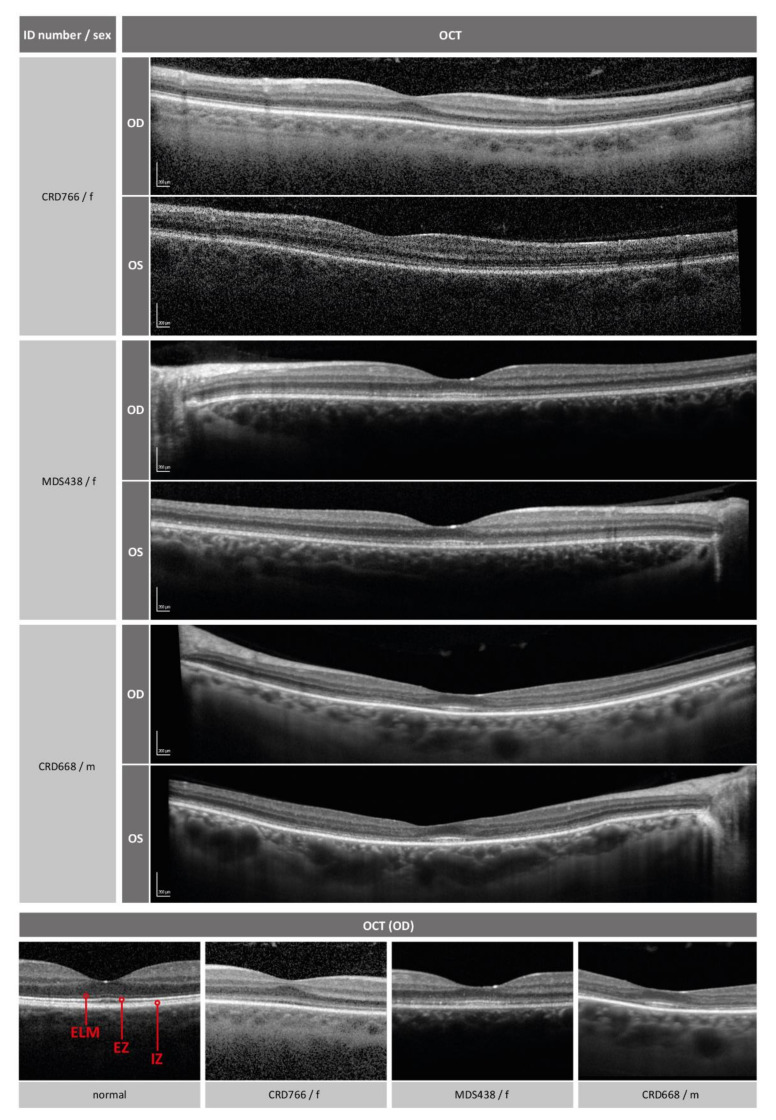
Visualization of retinal layers with optical coherence tomography (OCT). The lower row shows magnifications of the foveal region. For comparison, an example of a normal eye is shown on the left. ELM (external limiting membrane), EZ (ellipsoid zone) and IZ (interdigitation zone, outer segments) are indicated. The foveal region of the patients show diffuse disturbance of the ELM and EZ/IZ layers (CRD766, MDS438) or perifoveal atrophy of the layers with relative foveal sparing (CRD668). OD, right eye; OS, left eye; f, female; m, male.

The results obtained by multimodal clinical phenotyping are summarized in Figure 1, Figure 2, Figure 3. All three patients described here were adults (age 24, 27, 47, two females and one male). They reported nystagmus, reduced visual acuity, reduced contrast perception, photophobia and nystagmus as their first symptoms with age of onset ranging from early childhood (two years) to adulthood (22 years). The best corrected visual acuity (BCVA) at the time of examination was reduced in all three patients. All patients complained about photophobia, but not of night blindness, with the exception of the oldest subject who reported some recent difficulties seeing at night. Color vision defects were detected with hue tests only in the two oldest patients. The photopic responses on full-field electroretinography (ERG) were abnormal in all subjects, in contrary to the scotopic responses that were either normal or only slightly reduced (Figure 1). Corresponding to this, dark adapted full-field stimulus (FST) thresholds for blue and white were not pathologically increased. These findings indicate pronounced defects of cone function, but only small disturbances in rod function.

Retinal imaging revealed normal funduscopy and fundus autofluorescence in all patients (Figure 2). Disrupted photoreceptor structures were visible upon OCT. Figure 3 shows that the inner retinal layer as well as the outer nuclear layer of the photoreceptors were preserved in all patients. The external limiting membrane (ELM) was visible, but of blurry appearance. Furthermore, the EZ line, representing part of the inner segments and closely connected to the junction of inner segment and outer segment (IS/OS), was not clearly distinguishable. This effect was rather diffuse in the two younger patients and most pronounced parafoveally with a relative foveal sparing in the oldest patient. The retinal pigment epithelium as well as the Bruch’s membrane were well preserved in all patients.

### 2.2. Validation of Variants

All three patients in this study underwent diagnostic genetic testing by whole genome sequencing. Methodological details have already been published [13]. Briefly, sequencing (2 × 150 bp paired-end reads) was performed on an Illumina platform (NovaSeq6000). The average coverage on target ranged from 42× (patient CRD668) to 50× (patient MDS438). Variants were prioritized that were rare (minor allele frequency ≤0.01) and in known retinal disease genes. Putative pathogenic variants compatible with the phenotype were only identified in the *POC1B* gene (Table 1), namely a frameshift duplication variant in apparent homozygous state in patient CRD766, a canonical splice site variant and a deep intronic variant in patient MDS438, and a nonsense variant and a NCSS variant in patient CRD668, respectively. The variants in the latter two patients were found in heterozygous state. *Trans* configuration of variants could only be established in patient MDS438 by allelic cloning.

One of the two *POC1B* variants found in patient CRD668 is a nonsense variant (c.1340C > G/p.(S447 *)), resulting in a premature termination codon (PTC), while the other variant (c.561–3T>C) is located in the vicinity but outside the highly conserved GT splice acceptor dinucleotide. The effect of such a NCSS variant is difficult to interpret. Hence, according to the standards and guidelines provided by the American College of Medical Genetics and Genomics (ACMG) [14], the c.561–3T>C variant has to be classified as variant of uncertain significance (VUS) unless its effect on splicing has been validated.

The canonical splice site variant c.810+1G>T that was found in patient MDS438 has been described before and was shown to induce skipping of exons 6 and 7, or exon 7 alone [3]. In *trans*, we identified a rare variant in intron 9 (c.1033–327T>A), classified as VUS.

To evaluate the potential pathogenicity of the NCSS variant and the deep intronic variant, we performed an in silico analysis using two splice prediction algorithms. Conflicting results were obtained for variant c.561–3T>C which was predicted by SpliceAI to destroy the acceptor site of exon 6 with a Δscore of 0.21, while NNSplice predicted no effect. Concordant results were obtained for the c.1033–327T>A variant, which was predicted by both algorithms to create a novel acceptor site with a Δscore of 0.6 obtained with SpliceAI and a score of 0.61 obtained with NNSplice, respectively. Variant c.1033-327T>A was predicted by SpliceAI to also destroy the acceptor site of an alternative exon with a Δscore of 0.42. This alternative exon (here designated as exon 9a) is part of two minor Ensembl transcripts, which are predicted to be subjected to NMD (ENST00000547496.5), or to not be translated (ENST00000549304.5). A cryptic donor splice site with a score of 0.92 was predicted by NNSplice 28 nucleotides downstream of the cryptic acceptor splice site, thereby providing a potential complementary splice site that can be activated, possibly resulting in the inclusion of a pseudoexon in the mRNA. It is important to note that this pseudoexon differs from the alternative exon 9a by five nucleotides at the 5′ end (see Supplementary Figure S1).

In order to assess the effect of the c.561–3T>C and the c.1033–327T>A variant, we made use of heterologous splice assays (also often referred to as minigene assays) in human embryonic kidney (HEK)293T cells to test mutant and wildtype *POC1B* minigene constructs in direct comparison.

**Figure 4:**
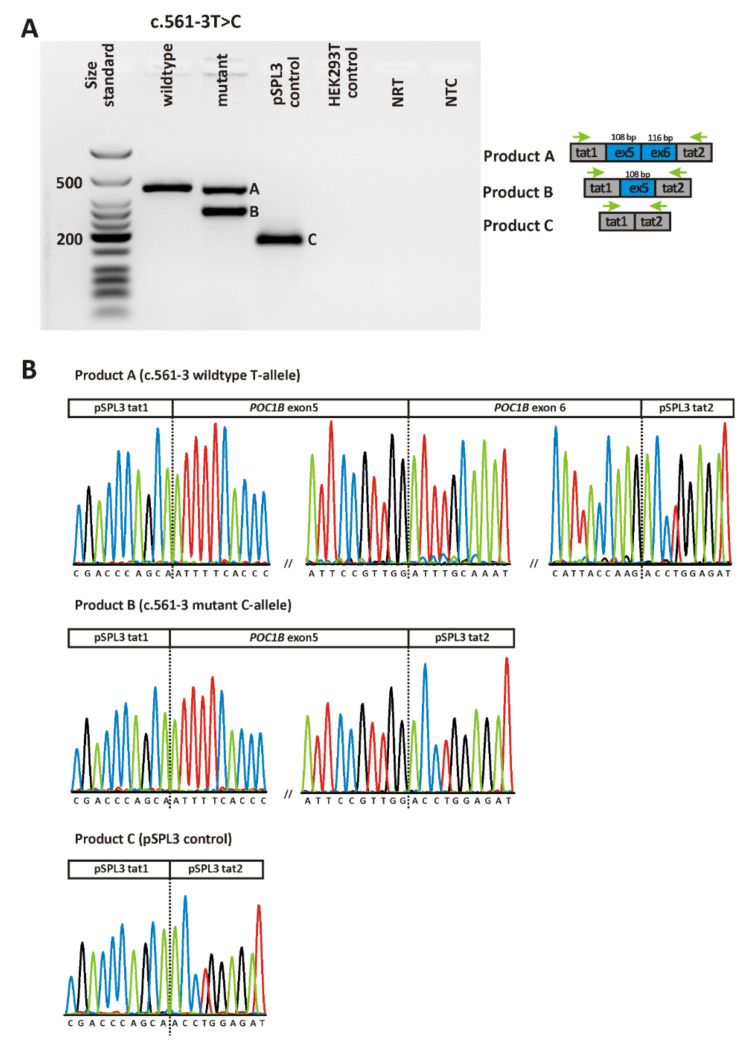
In vitro splicing assay for variant c.561-3T>C. (**A**) Agarose gel electrophoresis of RT-PCR products. Gel loading is as follows: A size standard (low molecular weight DNA ladder, NEB) is loaded in the leftmost lane. The RT-PCR product derived from HEK293T cells transfected with a plasmid construct harboring the wildtype allele is shown in lane 2, while the RT-PCR products obtained upon transfection with the mutant allele are shown in lane 3. RT-PCRs from transfection with empty pSPL3 vector (lane 4) and untransfected HEK293T cells (lane 5) served as controls. NRT (lane 6), no reverse transcriptase control; NTC (lane 7), no template control. Schemes of the amplified products are presented next to the agarose gel picture. Grey boxes represent pSPL3 resident exons tat1 and tat2, and blue boxes *POC1B* exons, respectively. The green arrows indicate the location of the RT-PCR primers. (**B**) Sequencing analysis of RT-PCR products. Sequencing analysis shows that the single RT-PCR product derived from transfection with the wildtype minigene construct corresponds to correct splicing (i.e., splicing of *POC1B* exons 5 and 6 between the pSPL3 resident exons tat1 and tat2), while the smaller transcript expressed by cells transfected with the mutant minigene construct shows skipping of exon 6. The bigger transcript corresponds to correct splicing (not depicted). Sequence analysis of the single RT-PCR product obtained from cells transfected with the “empty” pSPL3 plasmid shows that the two resident exons of the pSPL3 vector (i.e., tat1 and tat2) are spliced together.

**Figure 5:**
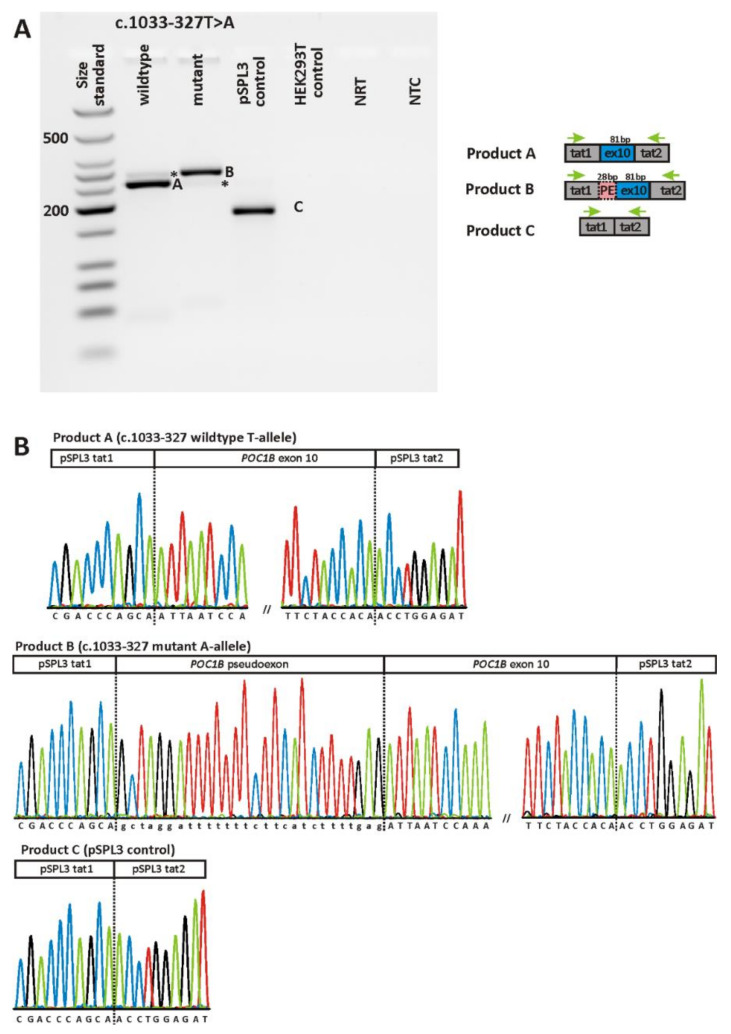
In vitro splicing assay for variant c.1033-327T>A. (**A**) Agarose gel electrophoresis of RT-PCR products. Gel loading is as follows: A size standard (low molecular weight DNA ladder, NEB) is loaded in the leftmost lane. RT-PCR products derived from HEK293T cells transfected with the plasmid construct harboring the wildtype allele are shown in lane 2, while those obtained upon transfection with the mutant allele are shown in lane 3. RT-PCRs from transfection with empty pSPL3 vector (lane 4) and untransfected HEK293T cells (lane 5) served as controls. NRT (lane 6), no reverse transcriptase control; NTC (lane 7), no template control. Schemes of the amplified products are presented next to the agarose gel picture. Grey boxes represent pSPL3 resident exons tat1 and tat2, and blue boxes *POC1B* exons, respectively. The green arrows indicate the location of the RT-PCR primers. Asterisks indicate products that could not be captured by subcloning. (**B**) Sequencing analysis of RT-PCR products. Sequencing analysis shows that the major RT-PCR product derived from transfection with the wildtype minigene construct corresponds to correct splicing (i.e., splicing of *POC1B* exon 10 between the pSPL3 resident exons tat1 and tat2), while the major transcript expressed by cells transfected with the mutant minigene construct shows the inclusion of a pseudoexon of 28 bp. Sequence analysis of the single RT-PCR product obtained from cells transfected with the “empty” pSPL3 plasmid shows that the two resident exons of the pSPL3 vector (i.e., tat1 and tat2) are spliced together.

Figure 4 and Figure 5 show the RT-PCR products obtained upon transfection of HEK293T cells with minigene constructs harboring either the respective wildtype or mutant allele for the two variants. RT-PCR was performed using primers binding to the vector resident HIV-derived exons tat1 and tat2 of the minigene construct. For variant c.561–3T>C, transfection with the mutant construct yielded two RT-PCR products, one of which being clearly smaller than the single product obtained from cells transfected with the wildtype allele (Figure 4A). Note that the gel image was taken with overexposure to exclude the presence of additional transcripts. Subcloning of RT-PCR products and sequencing of individual clones revealed skipping of exon 6 for the smaller transcript obtained upon transfection with the mutant allele. Skipping of exon 6 is predicted to result in a truncated protein (p.(F188Sfs*6)). The bigger transcript obtained upon transfection with the mutant allele was shown to represent the correctly spliced product (i.e., exon 6 spliced between exon 5 and the vector resident tat2 exon) (Figure 4B).

The variant located in intron 9, c.1033-327T>A, also exerted a splicing defect in the minigene assays. HEK293T cells transfected with the minigene construct harboring the mutant c.1033-327A-allele yielded a RT-PCR product which was clearly larger than the major product derived from cultures transfected with the wildtype c.1033-327T-allele (Figure 5A). Subsequent sequencing of the aberrant transcript revealed an out of frame pseudoexon of 28 nucleotides spliced between the canonical exons 9 and 10 (Figure 5B). This pseudoexon had exactly been predicted in silico (Supplementary Figure S1). The aberrant transcript would lead, if translated, to an insertion of eight novel amino acids followed by a PTC, hence the outcome is predicted to result in a truncated protein (p.(I345Afs*9)). Of note, strong overexposure revealed an additional RT-PCR product in the agarose gel which might reflect the correctly spliced transcript. However, this additional transcript could not be captured by subcloning. Conversely, the expression of the wildtype construct yielded also minor amounts of an aberrant transcript similar in size as the transcript harboring the pseudoexon. Note that the overexposure was necessary to make the additional bands visible but at the same time precluded quantification by densitometry (i.e., the assessment of the ratio between correct and aberrant transcript).

**Figure 6:**
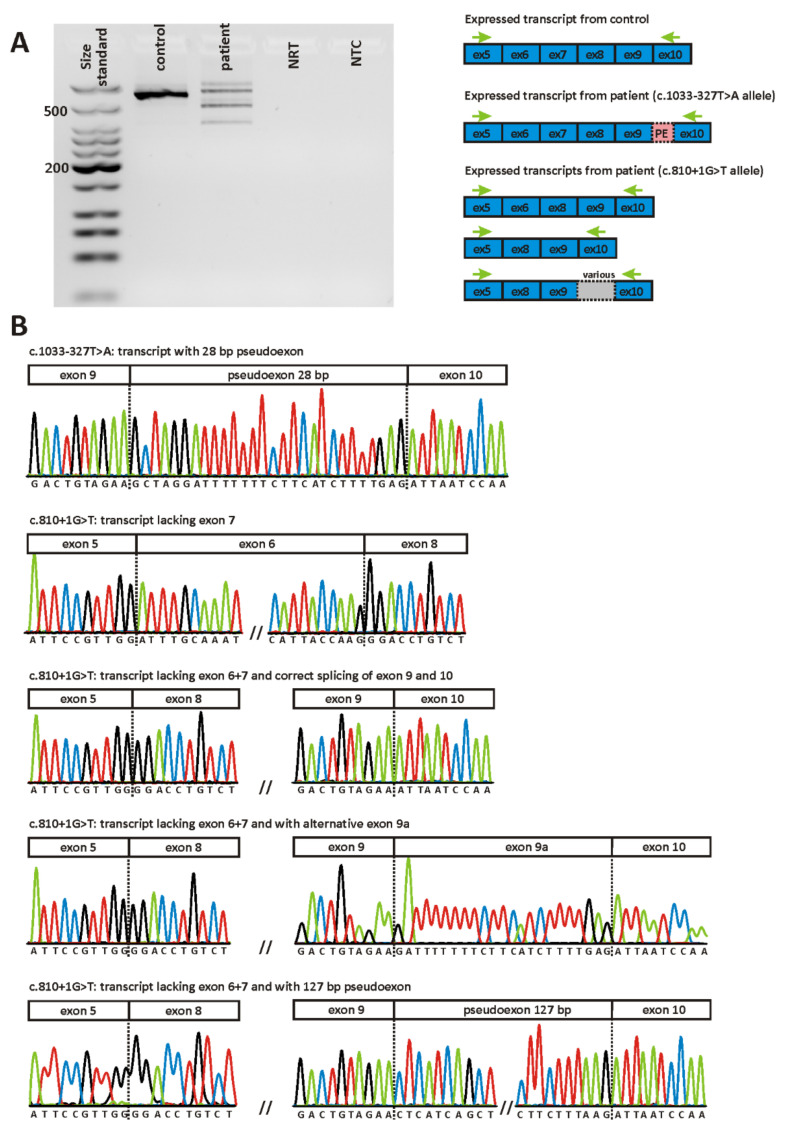
Direct transcript analysis in patient MDS438 from blood. (**A**) Agarose gel electrophoresis of RT-PCR products. Gel loading is as follows: A size standard (low molecular weight DNA ladder, NEB) is loaded in the leftmost lane. RT-PCR products derived from a healthy control person are shown in lane 2, while those from the patient are shown in lane 3. NRT (lane 4), no reverse transcriptase control; NTC (lane 5), no template control. Schemes of the amplified products are presented next to the agarose gel picture. (**B**) Sequencing of patient´s RT-PCR products following subcloning. Only relevant junctions are shown. Three major transcripts could be identified: Transcripts expressed by the c.1033-327T>A allele with a pseudoexon of 28 bp spliced between exon 9 and exon 10 and transcripts expressed by the c.810+1G>T allele lacking either exon 7 or both exon 6 and 7. In addition, some transcripts lacking both exon 6 and 7 showed additional aberrant splicing between exon 9 and 10: inclusion of the 28 bp pseudoexon (not depicted), inclusion of the alternative exon 9a or inclusion of a 127 bp pseudoexon.

As *POC1B* is expressed in peripheral blood cells, we aimed to confirm the splicing defects observed in the minigene assays using RNA isolated from a blood sample donated by patient MDS438. Agarose gel-based analysis showed that the RT-PCR reaction using patient-derived cDNA as template yielded several products (Figure 6A), all of which were quite weak when compared to the RT-PCR reaction from a healthy control person. This might be explained by the instability of both allelic transcripts due to nonsense mediated decay (NMD).

Since the pattern of different transcripts was too complex for a quantitative assessment by densitometry, we subcloned the RT-PCR from patient MDS438 and sequenced 100 individual clones (Figure 6B). Ninety-five percent of expressed *POC1B* transcripts displayed aberrant splicing while wild-type splicing was supported by 5% of transcripts. All of the aberrant transcripts detected result in a frameshift and thus are supposed to undergo NMD, consistent with the observed decrease in *POC1B* expression when compared to a healthy control. The majority of subclones were shown to lack exon 7 (40%) or both exon 6 and exon 7 (27%). These clones reflect transcripts that are expressed by the c.810+1G>T allele. Of note, eleven of the 27 clones that lacked both exon 6 and exon 7 revealed additional aberrant splicing events involving intron 9. Twenty-eight subclones (28%) did not show skipping of exon 6 and/or exon 7, but instead had a pseudoexon of 28 bp spliced between exon 9 and exon 10. Hence, these clones reflect the transcript that is generated by the c.1033-327T>A variant on the opposite allele. Five clones (5%) revealed a correctly spliced product.

## 3. Discussion

The three patients described in this manuscript present with clinical findings that are in line with those reported in previous publications [4,6,7,9,10]. All patients showed impaired cone function leading to reduced visual acuity, color vision disturbances, photophobia or nystagmus. However, the effect on rod function was more variable. Only two out of three patients showed impaired rod function with only a slight reduction in patient CRD668 and a more pronounced reduction in patient CRD766. Objective evaluations of cone function such as full-field ERG confirm this phenotype (Figure 1). In other publications, rod responses in the *POC1B* cohort were not recordable at the age of 61 years, and subnormal in younger adults [4]. Based on these observations, *POC1B*-associated retinopathy is mostly classified as either CD or CORD. Retinal imaging shows disrupted photoreceptor structure in the ELM, (IS/OS) junction, and the EZ, the latter representing the photoreceptor inner segment ellipsoid. This observation, described in previous reports [3,4,7,10] and confirmed by our study, is in concordance with the importance of POC1B for the maintenance of an intact ciliary structure. Similarly, a diffuse thinning of the outer retina is caused by the reduction of the above-mentioned retinal layers [9]. POC1B has been shown to localize at the basal body of the connecting cilium between the inner and outer photoreceptor segments [3]. Although POC1B seems to be equally important for the ciliogenesis and activity of both cones and rods [3], all patients in our study show a more pronounced impairment of cones. Due to the very slow progression of the disease combined with normal fundus appearance, decreased visual acuity and photophobia, patients harboring pathogenic variants in *POC1B* are frequently misdiagnosed with achromatopsia [3,10]. Another misinterpreted diagnosis of *POC1B*-associated retinopathy is occult macular dystrophy, as was also originally suspected in our case MDS438. The age of onset is rather variable in our small cohort, which might be due to the very slowly progressive disease course with little impairment in daily life in early years, and may also depend on the genotype. A broad range of age at onset in *POC1B*-associated retinopathy has also been reported previously [4,7].

Diagnostic genetic testing revealed putatively pathogenic variants in the *POC1B* gene in all three patients described in this study. The frameshift duplication variant c.1331_1332dup/p.(T445Rfs*10) in patient CRD766 generates a transcript with a PTC. Most transcripts harboring PTCs are predicted to be subjected to NMD, which is a cellular surveillance mechanism that recognizes PTCs in a transcript and, instead of translating such transcripts, targets them for degradation [15]. However, if PTCs occur within the last exon of a gene, as is the case for the c.1331_1332dup variant, they do not activate NMD but yield a stable mRNA that results in a truncated protein [16,17]. In *POC1B*, a nonsense variant has already been reported that is located further downstream than the c.1331_1332dup variant [6]. Hence, the C-terminus of the POC1B protein is likely crucial for proper protein function and we therefore consider the c.1331_1332dup variant to be pathogenic. This notion is further supported by the fact that this patient had the earliest onset of disease, the lowest visual acuity and the strongest reduction also in rod function.

The c.561-3T>C variant at the acceptor splice site of intron 6 has not been reported so far. It affected splicing in the minigene assay, with partial skipping of exon 6, but with the concomitant production of correctly spliced transcript that reached an amount roughly equal to that of the mutant allele. The transcript lacking exon 6 is predicted to result in a truncated protein (p.(F188Sfs*6)). We hypothesize that the c.561-3 C allele represents a hypomorphic allele that may be un-masked as being deleterious if occurring in homozygous state or in heterozygous state with another pathogenic allele. Hence, we conclude that the c.561-3 C allele represents a “leaky” splice variant and may explain the phenotype of patient CRD668, given that it is present in *trans* with the nonsense variant (c.1340C>G/p./S447*)) found in this patient. The observed “leakiness” of the c.561-3T>C variant might result in residual protein function and explain why the BCVA in this patient was still 0.4/0.5 at age 47. Notably, a non-canonical splice site variant in *POC1B* at the acceptor splice site of exon 3 (c.101-3T>G) has been analyzed in a previous study [12]. In line with our results, this variant was shown to induce skipping of the respective exon. However, in contrast to the c.561-3T>C variant analyzed in our study, the c.101-3T>G variant was predicted in silico to create a novel acceptor site which could be confirmed in vitro by the presence of a second aberrant transcript that included exon 3 and the terminal dinucleotide of intron 2 [12].

Our minigene assay for variant c.1033-327T>A demonstrated that constructs harboring the mutant A-allele lead to the insertion of a 28 bp pseudoexon between exons 9 and 10. The inclusion of this pseudoexon generates an aberrant transcript that would lead, if translated, to an insertion of eight novel amino acids followed by a PTC (p.(I345Afs*9)). Agarose gel-based analysis of the RT-PCR indicates that the c.1033-327T>A variant is fully penetrant, since an additional transcript (that might reflect correct splicing) was only visible upon strong overexposure. Since variant c.1033-327T>A was shown to be in *trans* with a canonical splice site variant (c.810+1G>T) in patient MDS438, we conclude that both variants together explain the phenotype in this patient.

Direct transcript analysis using blood cells from patient MDS438 revealed a more complex splicing profile compared to the minigene assay, owing to the presence of additional aberrant transcripts generated by the second allele in this patient. The canonical c.810+1G>T change is associated with skipping of exon 7 alone or of exons 6 and 7 [3]. It has been shown previously that some splice variants can change the order of intron removal, thereby causing a double exon skipping [18,19]. Our quantitative analysis of RT-PCR products confirmed the results of Roosing and colleagues who showed that transcripts lacking exon 7 were more frequent than transcripts lacking both exon 6 and exon 7 [3]. We also observed that both transcripts expressed by the c.810+1G>T allele (*n* = 67) were more frequent than those expressed by the counter-allele c.1033-327T>A (*n* = 28). This unbalanced transcript ratio might indicate that NMD is more efficient for the transcript expressed by the c.1033-327T>A allele, but could also simply reflect the fact that the ligation of fragments of various sizes in a single reaction often favors the ligation of smaller fragments (i.e., transcripts lacking exon 6 and/or exon 7). Of note, eleven of the 27 clones that lacked both exon 6 and exon 7 revealed additional aberrant splicing events involving intron 9. As whole-genome sequencing was performed for patient MDS438, we can rule out variants in intron 9 other than c.1033-327T>A (which is only present on the counter allele) to be responsible for these aberrant splicing events. Of the eleven clones that showed aberrant splicing in intron 9, seven had a pseudoexon of 127 bp spliced between exons 9 and 10. This pseudoexon is defined by cryptic splice sites with very high scores as predicted by NNSplice (1.0 for the cryptic acceptor site and 0.84 for the cryptic donor site, see Supplementary Figure S1). Three clones showed the very same pseudoexon of 28 bp which was found in the transcript expressed by the counter allele harboring the c.1033-327T>A variant. One clone had an alternative exon of 23 bp spliced between exons 9 and 10 which differs from the 28 bp pseudoexon only by five nucleotides at the 5´ end. The (cryptic) donor site of the 28 bp pseudoexon and the alternative exon achieves a score of 0.92 with NNSplice, but neither of the two acceptor sites is recognized by this algorithm (see Supplementary Figure S1). We hypothesize that transcripts lacking exon 6 and exon 7 promote the recognition of pseudoexons in intron 9, but we cannot explain the underlying molecular mechanism. We would like to emphasize that our observations were made in blood cells and HEK293T cells. Both cell types might lack *trans* splicing factors that are present in the retina and ensure correct splicing.

So far, only a few patients harboring *POC1B* variants have been described. This hampers the assessment of genotype-phenotype correlations. Currently, there is no evidence that certain variant types cause a more severe phenotype than others. Strikingly, the very same missense variant c.317G>C/p.(R106P) in homozygous state has been described in patients with features of a syndromic ciliopathy [5] and in patients with nonsyndromic CORD [3]. It has been hypothesized that genetic modifier factors could account for disease expression of this particular missense variant as severe syndromic ciliopathy in one family and as CORD in the other [5]. Our study is too small to derive any genotype–phenotype correlations. However, one of the variants (c.561-3T>C) we have identified proved not to be fully penetrant in the minigene assays. Residual protein function might be responsible for the comparatively well-preserved visual acuity in patient CRD688.

## 4. Materials and Methods

### 4.1. Patient Enrollment and Retrieval of Blood Samples

The patients in this study were recruited and clinically examined at the Eye Hospital, University of Tübingen, Germany. Genomic DNA of patients was extracted from peripheral blood using standard protocols. Samples from all patients were recruited in accordance with the principles of the Declaration of Helsinki and were obtained with written informed consent accompanying the patients’ samples. The study was approved by the institutional review board of the Ethics Committee of the University Hospital of Tübingen under the study numbers 349/2003V and 116/2015BO2.

### 4.2. Clinical Evaluation

Patients underwent ophthalmic examination including detailed medical history, best corrected visual acuity (BCVA) testing, kinetic perimetry (Octopus 900, Goldmann-III4e-Stimulus, Haag-Streit GmbH, Wedel, Germany), slit lamp examination, fundus examination and photography including fundus autofluorescence images, and full-field electroretinography (ERG) according to the ISCEV (International Society for Electrophysiology of Vision) standards. Color vision was examined by the Farnsworth and Lanthony D-15 test. In addition, patients were tested using OCT (Spectralis^®^ OCT/HRA, 55 degrees, Heidelberg Engineering, Germany).

### 4.3. Genetic Diagnostic Testing

Genetic diagnostic testing was performed by whole genome sequencing. Methodological details have already been published [13].

### 4.4. In Silico Analysis

The potential pathogenicity of the non-canonical splice site (NCSS) variants identified in this study was assessed using NNSplice [20] and the SpliceAI lookup tool from the Broad Institute (https://spliceailookup.broadinstitute.org/; accessed on 20 April 2021) using default settings.

### 4.5. In Vitro Splice Assays

In vitro splice assays were performed as described previously [21]. Briefly, genomic segments encompassing the variant of interest along with flanking sequences were amplified from patient genomic DNA using a proofreading polymerase and cloned into the pSPL3 minigene plasmid vector. Heterozygosity of patients for the variants of interest enabled co-amplification of the mutant and the normal allele and subsequent parallel cloning of the mutant and wildtype minigene constructs. Cloned genomic segments were 1376 bp for patient MDS438 (GRCh37/hg19 12: 89,852,810–89,854,185; corresponding to exon 10 and flanking intronic sequences), and 1586 bp for patient CRD668 (GRCh37/hg19 12: 89,864,882–89,866,467; corresponding to exon 5, intron 5, exon 6, and flanking intronic sequences), respectively. The resulting minigene constructs in their wildtype and mutant versions were used to transfect HEK293T/17 cells (ATCC^®^ CRL-11268™), which were then analyzed with respect to splicing of minigene-derived transcripts using RT-PCR. Primers for PCR amplification, cDNA synthesis, and RT-PCR are available upon request. Genomic coordinates given in this manuscript are based on the GRCh37 genome (hg19). Mutation nomenclature is in accordance with HGVS recommendations and based on GenBank accession NM_172240.3 with nucleotide one being the first nucleotide of the translation initiation codon ATG.

### 4.6. Direct mRNA Analysis

In order to assess the effect of the c.1033-327T>A variant on the *POC1B* transcript, total RNA was isolated from PAXgene blood samples obtained from patient MDS438 and from a healthy control subject according to the manufacturer’s protocol (Qiagen, Hilden, Germany). Four hundred ng of total RNA was used for cDNA synthesis using oligodT primers and the Maxima H Minus Reverse Transcriptase according to the manufacturer’s protocol (Thermo Fisher Scientific, Carlsbad, CA, USA). Reverse transcription polymerase chain reaction (RT-PCR) was performed using 2 µL cDNA, a forward primer located in exon 5 (5′-TTTTCACCCGATGGAAGACT-3′), a reverse primer located in exon 10 (5′-GCAAATCGATTACCTCAAGCT-3′), and standard PCR conditions (35 cycles). Subcloning of RT-PCR products was performed using the NEB^®^ PCR Cloning Kit according to the manufacturer’s protocol (NEB, Frankfurt, Germany). Plasmid DNA isolation was performed using the peqGOLD Plasmid Miniprep I kit (VWR International GmbH, Darmstadt, Germany) and sequencing was performed using Big Dye Termination chemistry (Applied Biosystems (ABI), Weiterstadt, Germany) and separation of sequencing products on a capillary sequencer (ABI 3100 genetic analyzer).

## 5. Conclusions

To conclude, we were able to validate two putative splice variants in *POC1B* by using minigenes, thereby changing their classification from VUS to pathogenic. We propose that minigenes represent a valid alternative to direct cDNA analysis if tissues expressing the gene of interest are not available. What is more, minigenes enable the assessment of transcripts generated from a single allele, thereby being superior to direct cDNA analysis which often cannot distinguish transcripts derived from both alleles. This approach continues to gain importance with the widespread use of genome sequencing in routine diagnostics, which identifies a large number of non-coding variants of uncertain significance. In addition, the multimodal evaluations of three cases with *POC1B* retinopathy expand the current clinical knowledge about this very rare phenotype of *POC1B*-associated slowly progressive CD or CORD.

## Figures and Tables

**Table 1:** *POC1B* variants identified in this study.

Patient	cDNA (NM_172240.3)	Protein (NP_758440.1)	Zygosity	Trans Config. Validated	gnomAD MAF	SpliceAI Prediction (0–1)	NNSplice Prediction (0–1)
CRD766	c.1331_1332dup	p.(T445Rfs*10)	hom	no	-	N/A	N/A
MDS438	c.810+1G>T	p.(V226Gfs*30) AND p.(F188Dfs*73)	het	yes	2.715E-05	Donor loss (0.99)	Wildtype donor not recognized
	c.1033-327T>A	p.(I345Afs*9)	het		4.789E-05	Acceptor gain (0.6)	Acceptor gain (0.61)
CRD668	c.561-3T>C	p.(F188Sfs*6)	het	no	-	Acceptor loss (0.21)	No effect
	c.1340C>G	p.(S447*)	het		-	N/A	N/A

MAF, minor allele frequency; hom, homozygous; het, heterozygous; config., configuration; N/A, not analyzed.

## Data Availability

The data that support the findings of this study are available from the Institute of Medical Genetics and Genomics (IMGAG) in Tübingen, but restrictions apply to the availability of these data, which were used under license for the current study, and so are not publicly available. Data are however available from the authors upon reasonable request and with permission of the IMGAG.

## Supplementary Materials

The following are available online at https://www.mdpi.com/article/10.3390/ijms22105396/s1.
