# Supplementary material for: Clinical Characteristics of POC1B-Associated Retinopathy and Assignment of Pathogenicity to Novel Deep Intronic and Non-Canonical Splice Site Variants
Source: Int J Mol Sci. 2021 May 20;22(10):5396. doi: 10.3390/ijms22105396 (PMC8160832; doi:10.3390/ijms22105396)
Supplement: Supplementary file 1 [file ijms-22-05396-s001.zip › ijms-1220857-supplementary.pdf]

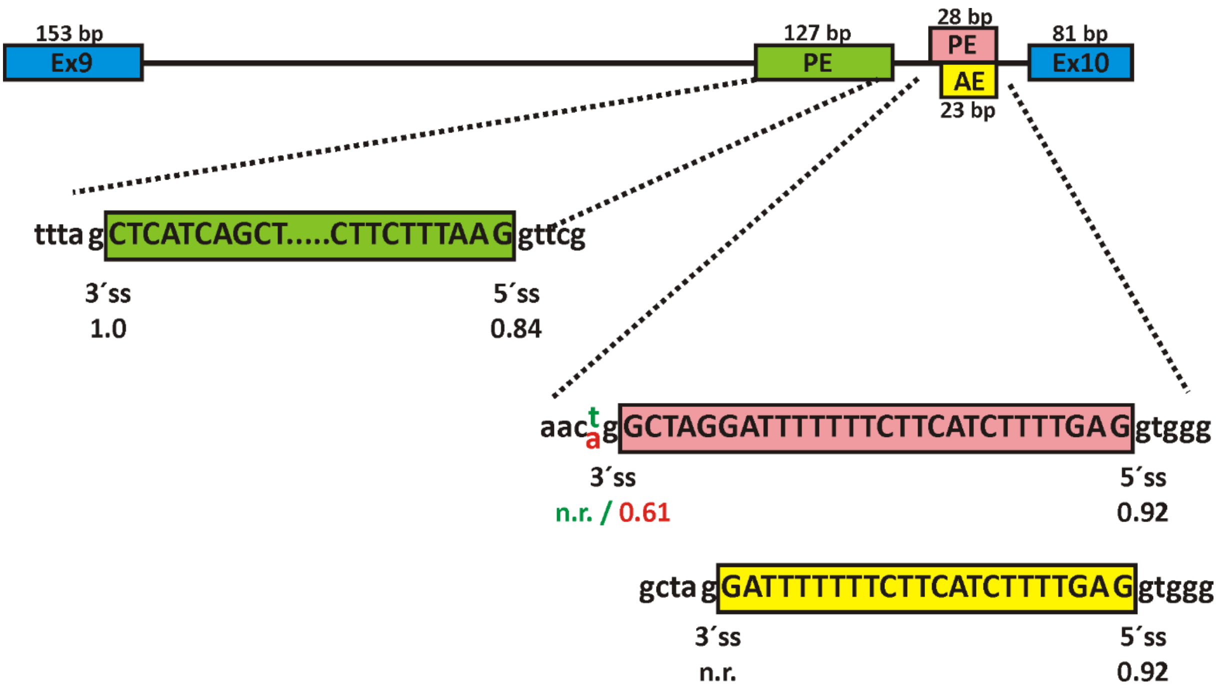

Supplementary Figure S1: Schematic representation of *POC1B* intron 9. Shown in yellow is an alternative exon (AE) of 23 bp which is part of two minor Ensembl transcripts that are predicted to be subjected to NMD (ENST00000547496.5), or not to be translated (ENST00000549304.5). Shown in pink and green are two pseudoexons of 28 and 127 bp, respectively. The bigger pseudoexon is defined by cryptic acceptor and donor splice sites with scores of 1.0 and 0.84, respectively, as predicted by NNSplice. The alternative exon and the smaller pseudoexon are defined by a cryptic donor site with a score of 0.92 as predicted by NNSplice. Neither the acceptor site of the alternative exon nor that of the smaller pseudoexon in its wildtype version were recognized by NNSplice. Only in case of the mutant c.1033-327A-allele does NNSplice predict a cryptic acceptor splice site with a score of 0.61. 3´ss, acceptor splice site; 5´ss donor splice site; n.r., not recognized by NNSplice.
